# Harm reduction in an emergency response to homelessness during South Africa’s COVID-19 lockdown

**DOI:** 10.1186/s12954-020-00404-0

**Published:** 2020-08-24

**Authors:** Tessa S. Marcus, Jan Heese, Andrew Scheibe, Shaun Shelly, Sasha X. Lalla, Jannie F. Hugo

**Affiliations:** 1 COPC Research Unit, Department of Family Medicine, School of Medicine, Faculty of Health Sciences, University of Pretoria, P/Bag x323, Arcadia, City of Tshwane, 0007 South Africa; 2 COPC Research Unit, Community Oriented Substance Use Programme, Department of Family Medicine, School of Medicine, Faculty of Health Sciences, University of Pretoria, P/Bag x323, Arcadia, City of Tshwane, 0007 South Africa

**Keywords:** COVID-19, Harm reduction, Homelessness, Opioid substitution therapy, National state of disaster lockdown, Emergency shelter

## Abstract

**Background:**

Caledonian Stadium, the main mass temporary shelter for homeless people in the City of Tshwane, was created as a local response to the imperatives of the novel coronavirus disease (COVID-19) National State of Disaster lockdown in South Africa. This is a case study of the coordinated emergency healthcare response provided by the University of Pretoria’s Department of Family Medicine between 24 March and 6 April 2020.

**Methods:**

This study uses a narrative approach to restory situated, transient, partial and provisional knowledge. Analysis is based on documented data and iteratively triangulated interviews on the operational experiences of selected healthcare first responders directly involved in the shelter.

**Results:**

The impending lockdown generated intense interactions by UP-DFM to prepare for the provision of COVID-19 and essential generalist primary with partners involved in the Community Oriented Substance Use Programme (COSUP). With approximately 2000 people at the shelter at its peak, the numbers exceeded expectations. Throughout, while government officials tried to secure bedding, food and toilets, the shelter was poorly equipped and without onsite management. The COSUP clinical team prioritised opioid substitution therapy using methadone and COVID-19 screening over generalist healthcare to manage withdrawal and contain tension and anxiety. COSUP and its partners helped the city plan and implement the safe re-sheltering of all Caledonian residents.

**Conclusion:**

The Caledonian shelter is an account of organisational resilience in the face of homelessness and substance use emergencies triggered by lockdown. Through community-oriented, bottom-up self-organisation, a clinically led team navigated a response to the immediate needs of people who are homeless and/or use drugs that evolved into a more sustainable intervention. Key lessons learnt were the importance of communicating with people directly affected by emergencies, the value of using methadone to reduce harms during emergencies and the imperative of including OST in essential primary healthcare.

## Background

COVID-19, as a novel, infectious disease, puts healthcare systems and healthcare service providers’ front and centre of preparing and responding to the pandemic. However, and notwithstanding the primacy of medical science and clinical care, public health management by definition requires interdisciplinary, intersectoral and interagency cooperation [1]. This requirement is amplified in disasters and emergencies that emanate from pandemics.

**Fig. 1:**
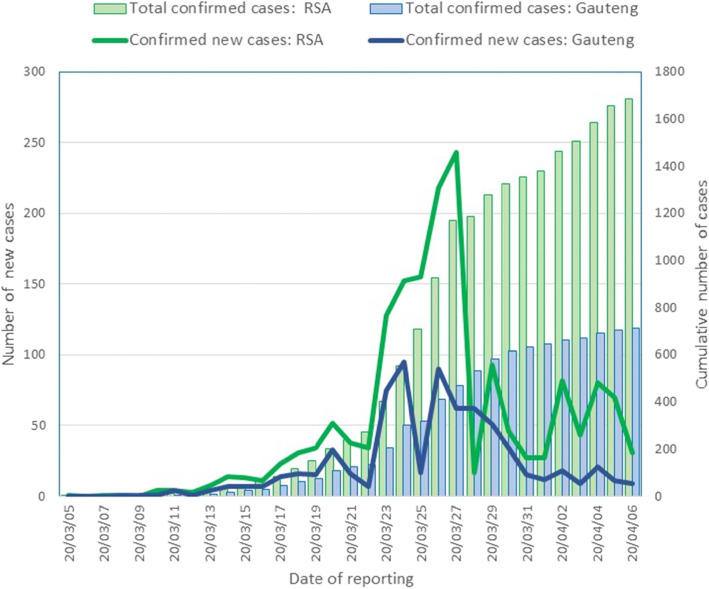
COVID-19 Republic of South Africa and Gauteng Province: confirmed cases and new cases (5 March–6 April 2020)

On 23 March 2020, the president of South Africa announced a 21-day national lockdown (26 March–16 April, extended to 30 April) [2]. It followed from government’s declaration of a national state of disaster on 15 March 2020 [3] that was triggered by the World Health Organisation’s (WHO) [4] characterisation of COVID-19 as a pandemic (11 March 2020) and the identification and spread of infection in South Africa (Fig. 1).

Of particular note regarding lockdown is Regulation Section 11D [5]. It simultaneously mandates the state to create temporary shelters for the homeless and authorises enforcement officers to forcibly evacuate people to them in order to preserve their own and others’ lives. It also mandates the provision of temporary sites to prevent transmission and enable treatment for people exposed to or infected with the new coronavirus who are unable to self-quarantine or self-isolate.

In the 3 days before lockdown, while media attention focused on the frantic scramble for essential supplies by people with available income [6], the announcement triggered an urgent effort to have a health and care response at municipal, district and facility level. Beginning 23 March 2020, the Department of Family Medicine at the University of Pretoria took several initiatives, including holding daily virtual COVID-19 pandemic response meetings to respond to the exigencies of sustaining essential primary healthcare in all projects and at all service sites.

Participation in COVID-19 Family Medicine daily meetings draws on the existing multidisciplinary, multisectoral core of expertise of faculty and staff as well as not for profit and municipal, district and national government personnel. All participants are involved in healthcare research, education and service delivery at the university as well as in district and municipal facilities and projects. Collectively they combine years of clinical experience and expertise in providing generalist integrated community-oriented primary care [7]. They also are involved in best practice harm reduction and substance use management through the Community Oriented Substance Use Programme (COSUP) [8] implemented with the City of Tshwane Metropolitan Municipality and Gauteng Department of Health’s Tshwane District (South Africa).

**Fig. 2:**
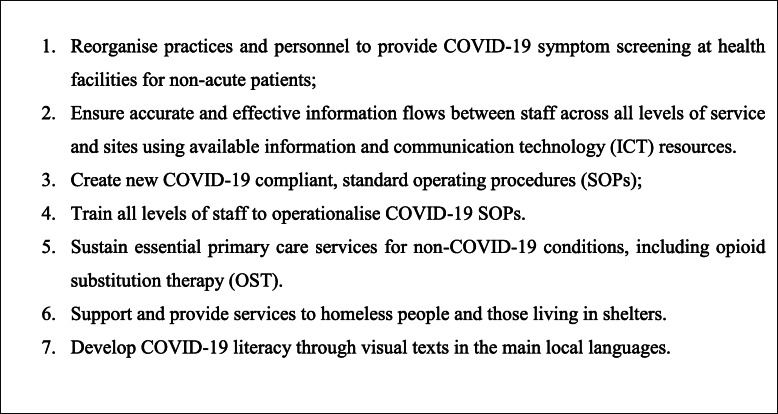
Seven priority COVID-19 action points in Tshwane

During the first COVID-19 daily meetings (23–26 March 2020) the team identified seven priorities for action (Fig. 2).

Although the provision of an integrated primary health, substance use management response was anticipated (Fig. 2: 5, 6) the need to create and provide services for people in temporary mass shelters was not foreseen. This is a case study of the Caledonian Stadium, the main mass temporary shelter created for homeless people in the City of Tshwane between 24 March and 3 April 2020.

The objective of the article is to provide a qualitative, firsthand empirical account of the response to an emergency that emerged from the announcement of the COVID-19 lockdown.

## Methods

Using a narrative approach [9], the content of the operational experiences of selected healthcare “first responders” is explored to gain an understanding of the immediate life-saving care that they and others provided and to uncover their continuous assessment of the nature and potential development of the emergency. Data are drawn from key informant interviews with three professionals directly involved in the organisation and delivery of COSUP services to the stadium population. Their accounts are supplemented by correspondence, meeting notes and memoranda, as well as standard operating procedures, guidelines, reports and official documents. In the process, situated, transient, partial and provisional knowledge was constructed into a narrative that makes sense of and creates order (chronological and categorical) about rapidly unfolding events [10–12]. Conversations and interviews with key informant experts were recorded or documented with the lead author and shared for review to ensure fact and portrayal accuracy. Primary documentary data (emails and WhatsApp™ messages) were sequenced and triangulated against interviewee accounts, official documents, internal and external reports and COVID-19 DFM daily meeting notes. Through repeated review of the data by the authors, the key elements of events were identified, organised and sequenced into a logical order and restoried.

The three key informant experts are (1) the lead clinician (a postgraduate doctor specialising in family medicine) who was responsible for Caledonian shelter clinical service delivery, health screening and opioid substitution therapy (OST) prescription; (2) a clinical associate (mid-level medical professional) who supported OST screening and daily dosing; and (3) the COSUP stakeholder liaison officer who engaged with key government, university and third sector organisations before and during the lockdown.

## Results

In the 3 days between the announcement of the lockdown and its implementation, COSUP and UP-DFM engaged in a series of meetings to plan a response to the lockdown. Internal meetings focused on identifying and working out how to render essential COSUP services. Meetings with partners focused on getting an understanding of the city’s departmental responsibilities and plans and, especially, the expectations of social development, the lead agency responsible for sheltering the homeless. Particular concern was raised about meeting needs and ensuring humanitarian treatment of vulnerable populations during the disaster [13].

On the evening before the lockdown (26 March 2020), small groups of people started arriving at the Caledonian Stadium. They had heard that “corona” was coming and that they would have to be off the streets by the next day. They said they had been advised to come to the shelter by city and other community workers. They came of their own volition, because they were scared and did not want to get sick. They also did not want to be locked up or sent “home” by the police [13, 14].

The stadium, a long neglected, city-owned facility without electricity and connected sewerage [15], was barely prepared. That evening, when the acting Provincial Member of the Executive for Social Development (Gauteng Province) arrived on site, provincial tents had been offloaded but not erected and there were water trucks and chemical toilets, but no lighting, mattresses, blankets or personal hygiene kits. Furthermore, there was no site management in place, although there was a small municipal police presence [13].

While some people continued to drift in throughout the night, by the morning (27 March 2020) they were arriving in significant numbers. Many walked from shelters, parks and derelict building “homeless hotspots” around town. On that first day of the lockdown, the South African Police Service^1^ also began dropping off people whom they found walking to the stadium from the suburbs and townships around the city [13].

Tents were being set up and, although the bedding was ordered by city officials, no mattresses and only about 240 blankets arrived. No one took charge of ensuring that the shelter was organised to address COVID-19 public health requirements, manage resident intake or inform people about what to expect and do. The scale of the influx of people was completely unanticipated [13].

As the day progressed, people with opioid dependence became increasingly agitated as they struggled to deal with withdrawal [13, 16]. To respond to an untenable situation, the Head of the Department of Family Medicine undertook to lead a coordinated district, municipal and not-for-profit healthcare response through COSUP in Tshwane [17]. A clinical team was redirected to the stadium, while COSUP continued to provide essential services at pre-existing sites that included moving stable OST clients from daily observed treatment to weekly take home dosing and initiating a mobile OST service [18]. In anticipation of increased need to manage withdrawal, they also obtained permission from the city to procure additional methadone [19].

Early on Saturday morning (28 March 2020), a small COSUP clinical team created a make shift service point outside the stadium. With the help of the municipal police, people who were withdrawing or in need of medical care came forward. For each person, the clinical team did a health intake and general substance use screening, followed by a consultation. Clients with a history of opioid use and clinical signs of withdrawal (as measured on the clinical opiate withdrawal scale) were given low initiation doses of methadone (20–30 mg) without baseline urinalysis. They wrote scripts, fetched medicines and administered directly observed treatment for those in need of methadone. Equipped with surgical masks, gloves and hand wash, they worked “on the fly” through a seemingly never-ending queue of people crushing up against the gazebo [16]. Municipal police broke up fights and helped keep order, and the city community development workers helped disinfect the spoons used to administer methadone. Social development personnel were present, albeit observing from their cars. Several had expressed their opposition to a harm reduction approach to substance use [14, 16]. At the end of 7 h of uninterrupted service, the team debriefed, shared out their hand-written records and went home to capture the data onto an electronic spreadsheet for use in the morning.

After considerable effort on the part of the Tshwane Homelessness Forum [20], the city head of Social Development convened a Homelessness Task Team with them, COSUP and relevant divisional management representatives [13]. The task team adopted the provincially endorsed COVID-19 shelter plan developed by the Tshwane Homelessness Forum and formalised COSUP’s role in COVID-19 screening, OST treatment and intake registration [13].

On the 29th of March, the stadium remained unmanaged. The numbers of people fluctuated, with an ebb and flow of people around meal time and COSUP clinical services. There was considerable overcrowding; people were not assigned places to sleep; there were no visible handwashing facilities; and the few toilets that were there were not being adequately serviced. Also, there was no place to separate anyone with respiratory symptoms, including those with TB [21].

After a family medicine operational review of COSUP sites [13], the Caledonian clinical team was reinforced by three post-graduate family medicine registrars. Armed with additional human resources and a better understanding of the tasks of the day ahead, the COSUP clinical team created two service stations. They split the queue between people who had been given methadone the previous day and those who needed to be screened or required other services. While they queued inside the stadium gates, each person completed a form to indicate the service they wanted [13, 16].

A number of Caledonian residents voluntarily assisted with patient flow, releasing people in groups of 10 and arranging chairs for them to wait to see a doctor or receive their supervised methadone dose. Together with city community development workers, they also supported physical distancing and handwashing at the stations [16].

Although the start of services was delayed until municipal police arrived to maintain crowd control, the COSUP stadium clinical team was able to get through the queue by the end of the afternoon. By obtaining methadone in sufficient quantities to meet demand, they were able to significantly reduce the time spent going back and forth to the pharmacy to fill scripts. Also, the use of measuring cups made dosing easier and quicker [14].

In response to the SAPS Joint Operations Command directive that all homeless people were to be in shelters and that they would assist by supporting the process of shelter creation, COSUP and the Tshwane Homeless Forum advised on the size and composition of other temporary shelters, finalised protocols on intake and screening and mobilised partner organisations (Harmless and the Foundation for Professional Development) to provide additional clinical support [13, 22].

On Monday, 30 March, media reports of dire conditions and disgruntlement at the shelter [23], led to a city management decision to enforce access control and restrict movement to better comply with the lockdown regulations [16]. At the time, there were between 1800 and 2000 people residing there.

The Caledonian clinical team continued to provide OST services outside the main gates and were able to meet the daily demand for methadone, even though many people were coming into the stadium from outside and several were trying to double dose. On site, with assistance from a Tshwane Homelessness Forum pastor to attend to people’s welfare needs, the COSUP team became the de facto face of care. The team, however, was still too small and lacked the space and resources to attend to people’s other physical and mental health needs. They were unable to provide a full harm reduction package of care. Harmless, the organisation with the largest needle and syringe service in the city was asked to hold off as municipal and national police were actively confiscating needles and the city regarded the concurrent provision of needle and syringe services and OST as a form of mixed messaging. There also were no social development personnel to assist with basic social services and family contacting [16, 21].

COSUP and other Homelessness Task Team members spent the day working with partners to identify alternative COVID-19 shelters. Although many public, private, faith-based and non-governmental organisations wanted to assist, few were able to comply with the public and personal health stringencies of COVID-19. Two immediately available sites were identified, and the team worked through the night to set them up as shelters [13].

On the 31st of March, the COSUP clinical team was instructed by the city to deliver services inside the stadium to prevent "walk-ins" off the street accessing OST. The clinical team felt unsafe to set up stations inside, as over the previous days they had observed municipal police personnel to be insufficiently trained to deal with crowds [16]. The metro police did not arrive and the person responsible for the stadium management did not respond to their request for security and crowd control assistance [13].

The planned staggered re-sheltering of people at the Caledonian and other congested shelters also began that day. The first group of 431 men and women who were not receiving OST were bussed to two new shelters. The remainder were re-sheltered in batches over the next 6 days at newly created, smaller facilities (Table 1).

**Table 1:** City of Tshwane COVID-19 emergency re-sheltering 31 March–6 April 2020

Date	Facility name	Capacity	Number placed	Sex	Age category	Status
31 Mar	Lucas van der Berg Stadium	**400**	400	M	Adults and youth	Homeless
31 Mar	Lyttleton Town Hall	**36**	31	M + F	Adults	Homeless
1 Apr	Oosterlig	**20**	20	M	Older adults	Homeless
1 Apr	NG Arcadia Church	**20**	20	M	Older adults	Homeless
1 Apr	Melodi Ya Tshwane	**25**	25	F	Adult mothers and their children	Homeless
3 Apr	Capital Park	**51**	52	M	Adults and youth	Homeless
3 Apr	Life Changing Ministries	**90**	90	M	Adults and youth	Homeless + PWUD
5 Apr	St Wilfreds	**24**	24	M	Adults and youth	Homeless
5 Apr	Mabopane Indoor Sport Centre	**150**	85	M	Adults and youth	Homeless + PWUD
6 Apr	Lyttelton Sports Park	**300**	348	M	Adults and youth	Homeless + PWUD
**Total**		**1116**	**1095**			

*PWUD* people who use drugs

Source: Authors’ own table

From 1 April onwards, the Caledonian clinical team delivered OST and other services from within the stadium. Space had been provided, but municipal police were only present at the beginning of the day and it was hard to create an orderly flow. These sub-optimal working conditions were made worse by a drop in temperature and insufficient tents; people were sleeping in groups at the entrance to shield themselves from the wind [24].

At the same time, the COSUP team involved in procuring methadone had to overcome pockets of bureaucratic resistance to its use for withdrawal management as well as difficulties in accessing it in sufficient quantities to meet demand [25].

On 2 April, the team saw 31 new clients. Most had been brought to the stadium by the police the previous evening, despite a directive to not do so. In the face of long queues, poor environmental and COVID-19 compliance at the site, and the impossibility of attending to the general health and wellbeing of residents, the COSUP clinical team urged that the process of re-sheltering be speeded up [21].

On 4 April, heavy rains flooded tents and washed people out of their sleeping places. The clinical team was forced to provide healthcare services to cold and drenched residents in a crowded narrow passage [16]. Over the next day and a half, the remainder of Caledonian residents were moved to alternative sites and the shelter was closed on 6 April.

During the period of re-sheltering, COSUP and the University of Pretoria’s Department of Family Medicine began to provide integrated primary healthcare at the 24 (8 permanent, 16 temporary) shelters in the city. Over and above providing OST to manage withdrawal, clinical care returned to an integrated community-oriented primary care approach to address acute and chronic conditions including infections, injuries, TB, HIV and mental health as well as the potential threat of COVID-19 in the homeless and substance-using populations [26]. However, needle and syringe service delivery was not permitted.

## Discussion

The Caledonian Stadium emergency shelter is the outcome of an emergent emergency [27]. Arising rapidly and unexpectedly from complex, often unpredictable processes, the emergency was triggered by the decision to lockdown the country to contain COVID-19. Notwithstanding ministerial, National Department of Health and WHO Africa Office efforts to mobilise government COVID-19 preparedness in the 6 weeks before the declaration of a National State of Disaster [28], the challenges of responding to the combination of homelessness and dependence on substances was neither foreseen nor planned for. Unlike preparation that is always anticipatory and hypothetical, this is not exceptional in emergencies, because responding to them is concrete, material and immediate [27].

This account tells a story of bottom-up self-organisation. Amid a chaotic and rapidly evolving environment, a network of organisations and actors coalesced to respond to the emergency through an effort to provide shelter, food and healthcare.

Among the many differences in focus and priorities, the needs of people with opioid dependence took centre stage. In part, this was because of the imperative of managing the unpleasant physical, mental and social side effects of withdrawal from opioids, including elevated temperature, increased respiratory rate, anxiety, nausea and diarrhoea, tachycardia, hypertension, sweating, tearing, runny nose and bone and muscle pain. Given many similarities with COVID-19 symptoms, it was important to ensure that withdrawal was not confused with or masked novel coronavirus infection. There also was a need to prevent the risk of fatal overdose, given the known loss of opioid tolerance even after a relative short period of abstinence [29].

Many lockdown responders were opposed to COSUP’s harm reduction approach to substance use and the need for OST. They had no grasp of the scale and physical effects of opioid dependence nor were they able to propose any practical options for the hundreds of people struggling with withdrawal inside and outside the stadium. In contrast, by providing methadone, COSUP’s clinical interventions reduced volatility and tension at the shelters and prevented fatal overdoses among clients across the city. The provision of methadone also served as a way into harm reduction, even though the emergency nature of the situation made it impossible to provide several essential harm reduction components, follow standard OST protocols or provide integrated HIV, TB, viral hepatitis or other chronic disease detection, treatment adherence and support.

Rendering a focused heathcare service alongside safely re-sheltering people in the midst of the lockdown required the deliberate nurturing of established relationships and the mobilisation of cooperation and collaborative practices among key organisations and individuals. Through a steady flow of communication, a diverse network of actors and skills were mobilised to respond to problems as they arose.

However, the critical issue of communication with the people living in the shelter was overlooked entirely. This added to the levels of anxiety and uncertainty among shelter residents, since they were not informed about the work being done, the challenges being faced or the plans being made. Also, even though several individuals came forward of their own volition to help, the population itself was insufficiently informed about or mobilised to protect their health and the health of others.

More generally, by demonstrating an effective response to street homelessness and substance use disorder under exceptional conditions, the Caledonian stadium experience made evident to policy makers and practitioners the possibility and necessity of providing shelter and healthcare services to homeless people and people who use drugs in “normal” times. As a consequence, a combined comprehensive approach to deliver services to these populations is being planned for long-term implementation by the city, the university and the relevant partner organisations.

The Caledonian Stadium story also talks to the portentous challenges of public and individual health in the COVID-19 pandemic. COSUP and other partners repeatedly warned of the inadequacy of the setup, layout and provision of essential utilities to ensure basic public health. Although there were no instances of COVID-19 among residents or healthcare providers, this was due mainly to chance and the stage of the epidemic in the city during lockdown. Had the epidemic been more advanced, the fact of the matter is that the creation of a poorly managed, inadequately prepared mass shelter would have been the perfect petri dish to culture the pandemic in the city. Equally, had the Department of Family Medicine and its partners not been able to surmount the multitude of bureaucratic, policy and personal belief barriers to providing methadone to manage withdrawal and prevent heroin overdose, the emergent emergency could have become a socially explosive, personal disaster for thousands of vulnerable city residents.

## Limitations

This account of the first 2 weeks of work has several limitations. By definition, it is partial both in terms of perspective and in time. The voices of other responders as well as the people coming in and outside the shelter would enrich the narrative. The story, too is still unfolding as the lockdown and the pandemic are far from over. Lastly, in the immediacy of the response data inevitably are partial and in places inconsistent, but the experience, threads and lessons override these gaps.

## Conclusion

The story of the Caledonian shelter is an account of organisational resilience in the face of a dual emergency around shelter and substance use that was triggered by the lockdown on 27 March 2020. Over 2 weeks, the Department of Family Medicine, through COSUP, navigated a clinically led, bottom-up, collaborative response to address the immediate needs of people who are homeless and may or may not use drugs in the City of Tshwane. Time, resources and urgency all combined to compress services in the emergency warp, until things were more stabilised. Through this response to contain the disruptive potential of an emergent emergency, a better, more sustainable COVID-19 intervention evolved. The key lessons learnt were the importance of communicating with people directly affected by emergencies, the value of using methadone to reduce harms during emergencies and the imperative of including OST in essential primary healthcare.

## Recommendations

1. Homelessness and harmful substance use interventions require a harm reduction approach that is bottom-up, inclusive, collaborative and multisectoral.
2. OST and related harm reduction services need to be enabled and applied.
3. It is critical to foreground communication with people directly affected by emergencies as well as those responding to them.
4. Best practice clinical therapy in opioid dependence, including in emergencies, needs to enable and apply OST and related harm reduction services.
5. The provision of essential comprehensive mental and physical healthcare for vulnerable populations during and beyond the COVID-19 pandemic is an imperative.

## Data Availability

All data generated or analysed during this study are included in this published article.

## Abbreviations

COSUP: Community Oriented Substance Use Programme
WHO: World Health Organisation
OST: Opioid substitution therapy

## Footnotes

1 Municipal police services are employed by local municipal authorities and are responsible for traffic policing, policing of municipal by-laws and regulations and preventing crime. The South African Police Service is a single national policing service responsible for crime prevention, detection and enforcement.
